# Chemical compositions, fatty acid profiles and selected contaminants in commercial potato and corn chips sold in the Tunisian market

**DOI:** 10.1007/s44187-022-00030-8

**Published:** 2022-10-31

**Authors:** Haifa Benkhoud, Yassine Mrabet, Nadia Nasraoui, Wided Bellazreg, Faten Daly, Najet Chaabane, Karim Hosni

**Affiliations:** 1 Institut National de Recherche et d’Analyse Physico-Chimique (INRAP), Laboratoire Des Substances Naturelles, Biotechpôle de Sidi Thabet, 2020, Ariana, Tunisia; 2 Institut National Agronomique de Tunisie, Université de Carthage, 1082, Tunis, Tunisia; 3 Institut National de Recherche et d’Analyse Physico-Chimique (INRAP), Unité Spécialisée de Développement et de Valorisation des Techniques d’Analyse, Biotechpôle de Sidi Thabet, 2020, Ariana, Tunisia

**Keywords:** Chips, Nutritional quality, Fatty acid, Safety, Acrylamide, Contaminants, Chemical composition

## Abstract

In the last decades, the snack food market is experiencing an important expansion due to the new fast-paced life-style associated with modernization. Crispy snacks, or chips are the most popular snack around the world, nevertheless, their overconsumption is related to the incidence of many diseases. Subsequently, this work aimed to study the chemical composition of 13 brands of potato and corn chips available in the Tunisian market. The investigation was based on: the determination of their chemical, mineral, and fatty acid composition; an evaluation of their lipid quality indices; and a chromatographic analysis of pesticides, aromatic hydrocarbons and acrylamide content. The results showed that the chips samples were of a high carbohydrate and fat content of up to 64.54% and 42.98%, respectively, versus a low protein and mineral composition. The fatty acid profiling showed that saturated fatty acids represent more than 39% for the majority of samples. A poor lipid quality was also observed through their low unsaturated fatty acids /saturated Fatty Acids ratios, with a mean value of 2.24 and their high atherogenic and thrombogenic indices that reached 1.69 and 2.23 respectively. While the analysis of pesticide residues and polycyclic aromatic hydrocarbons showed that all compounds were below the maximum allowed levels, the acrylamide analysis highlighted contamination in all the samples tested, with some values exceeding the allowed threshold. In conclusion, it can be suggested that the crisps sold in the Tunisian market are of a poor nutritional quality and they are potentially unsafe for human consumption.

## Introduction

The snack market was valued at 493.4 billion dollars in 2020 and is expected to grow to 732.6 billion dollars by 2026 [1]. The considerable increase of global demand for snack food was driven by the changes in lifestyle and eating habits affected by rapid urbanization and exacerbated recently with the Covid-19 pandemic lock-down [2]. The consumption of snack foods is widespread due to their specific sensory attributes, attractive shape, taste, packaging, and their crunchy texture [3]. Among the wide assortment of commercial snack foods are: potato, tortilla, multigrain, and corn chips, ready-to-eat popcorn, nuts and granola, etc. [4].The average volume per capita of the worldwide market in potato chips is estimated to be 2.1 kg in 2022, while tortilla chips and pretzels are estimated at 1.1 kg [5]. Despite its popularity, mounting evidence suggests that snack overconsumption was positively correlated with a positive energy balance, obesity, type-2 diabetes, hypertension, dental caries, periodontal and cardiovascular diseases [6]. These unhealthy factors were primarily attributed to the high fat and sodium content versus low fibre, vitamin, mineral and protein composition of snack food [7]. Several studies conducted on the chips’ fatty acid profile highlighted the high saturated fatty acid content, particularly that of C16:0 and the high isomeric trans fatty acid percentage [8, 9]. An overconsumption of trans fatty acids is known to lead to hypercholesterolemia and changes in the blood lipoprotein profile [10, 11]. Additionally, the use of preservatives and artificial flavours in their formulation, and the possibility of the generation and/or the introduction of some contaminants during the production process, such as acrylamide and polycyclic aromatic hydrocarbons (PAHs), may make them particularly genotoxic [12].

Despite the absence of a representative national survey, a tremendous increase in snack consumption has been observed in the Tunisian population. These trends, along with the increase in market demand for snack foods, have contributed to the establishment of several factories and the importation of many international brands. Surprisingly, even with the huge expansion of snacking in Tunisian society, data on their chemical composition and/or health attributes are lacking. Therefore, the aim of this study was to thoroughly investigate the chemical composition and safety of potato and corn chips available in the Tunisian market. The investigation was conducted on 13 national and international brands of potato and corn chips randomly selected from the local market. The study comprised the determination of the chemical composition, including: lipid, ash, carbohydrate, protein, and mineral content, fatty acid profiles and the nutritional quality of the chips. Additional analyses were conducted to identify chemical contaminants like acrylamide, PAHs and pesticide residues.

The results were expected to inform Tunisian consumers and official control bodies regarding the nutritional quality and safety of the most popular consumed chips. This data would provide a detailed overview of quality and safety of chips in Tunisian market which could be compared to other markets and served as a benchmark for designing novel functional snacks. In addition, it could inform health professionals’ interventions/recommendations to promote healthy snack foods as an alternative. Finally, it could provide a potential for the development of new snack foods with an improved nutritional quality and health attributes.

## Materials and methods

### Chips

A total of 13 different pre-packaged potato and corn chips samples from three different origins (Tunisia, Egypt and USA) were purchased from local markets in February 2018. The chosen samples, along with the codes and country of origins, are shown in Table 1.

**Table 1:** Chips brands used in this study

Chips name	Code	Supplier/Country
Potato chips with cheese	PTC1	Tunisia
Potato chips with Sour cream and oinion	PTC2	USA
Corn chips with cheese	CC1	Tunisia
Potato chips with Paprika	PTC3	Tunisia
Potato chips	PTC4	Tunisia
Potato chips with cheese	PTC5	Egypt
Potato chips With salt	PTC6	Tunisia
Corn chips with salt	CC2	Tunisia
Corn chips with cheese	CC3	USA
Potato chips	PTC7	Tunisia
Corn chips with salt	CC4	Tunisia
Corn chips with salt	CC5	Tunisia
Potato chips With salt	PTC8	Tunisia

### Samples preparation and analytical procedures

#### Chemical and elemental analysis

The chip samples dry matter was achieved by drying samples at 105 °C for 24 h until constant weight was obtained. Crude protein content was determined by the Kjeldahl method (N × 6.25). Total carbohydrate composition was determined by the method of Dubois [13]. Total lipid content was determined using a Soxhlet apparatus and hexane as an extracting solvent. Ash content was determined by incineration in a furnace oven at 550 °C for 6 h. Mineral content was ascertained using an inductively coupled plasma-atomic emission spectrometry ICP-OES [14].

#### Fatty acids composition

Fatty acid composition of the chips samples was determined by gas chromatography after methylation with 3% sodium methoxide (NaOCH_3_) and sulphuric acid [15]. The fatty acid methyl esters (FAMEs) were analyzed on a Hewlett-Packard 6890 gas chromatograph (Agilent Technologies, Palo Alto, California, USA) equipped with a flame ionisation detector (FID) and an electronic pressure control injector (EPC). They were separated on a TR-FAMEs capillary column (60 m × 0.25 mm, 0.25 μm film thickness). The column temperature was programmed as follows: isotherm at 100 °C for 5 minutes, then increased to 240 °C at 4 °C/min and held for 15 minutes. Temperatures of injector and detector were 240 and 260 °C, respectively. The carrier gas used was helium at a flow rate of 1 mL/min. The chromatographic peaks were identified by comparison of their retention time with those of authentic 37 standard FAMEs (Sigma-Aldich; Steinheim, Germany). The relative percentages of the different fatty acids were automatically determined by the CHEMSTATION software with reference to total fatty acids from the integrated FID pick areas.

#### Lipid quality indices

In addition to saturated fatty acids (SFAs), unsaturated fatty acids (UFAs), UFA/SFA ratio and ɷ3/ɷ6 ratio were determined. For the nutritional lipid quality, the following parameters including atherogenic index (AI), thrombogenic index (TI) [16], calculated oxidizability value (Cox) and the oxidative susceptibility (OS) as indicators of the nutritional lipid quality were calculated based on the FAMEs results:

$$\mathbf{A}\mathbf{I}=\frac{(\left(4\times \mathrm{C}14:0\right)+\mathrm{C}16:0+\mathrm{C}18:0)}{\mathrm{\Sigma UFA}+\mathrm{\Sigma \omega }6\mathrm{ PUFA}+\mathrm{ \Sigma \omega }3\mathrm{PUFA}}$$

$$\mathbf{T}\mathbf{I}=\frac{(C14:0+C16:0+C18:0)}{(0.5 \times \mathrm{MUFA}+0.5\times\upomega 6\mathrm{PUFA}+3\times\upomega 3\mathrm{ PUFA}+\frac{\upomega 3}{\upomega 6}\mathrm{PUFA}}$$

where MUFA is the sum of monounsaturated fatty acids, and PUFA is the sum of polyunsaturated fatty acids.

**                                                               Cox** = [C18:1 + 10.3 C18:2 + 21.6 C18:3]/100 [17]

**                                                               OS** = MUFA + 45 C18:2 + 100 C18:3 [18]

#### Gas chromatography analysis of acrylamide

For the determination of acrylamide concentration, pulverized chip samples were mixed with water, then homogenized, centrifuged and then subjected to a florisil SPE cartridges preconditioned with methanol and water, and eluted with water.

For the derivatization procedure, the SPE eluate was mixed with the bromination reagent and left to stand for 1 h in an ice bath. Then an aqueous solution of 1 M sodium thiosulfate (Na_2_S_2_O_3_) was added and the brominated acrylamide was extracted using ethyl acetate and dried over anhydrous sodium [19].

Brominated acrylamide was analysed using a a Hewlett-Packard 6890 gas chromatograph (Agilent Technologies, Palo Alto, California, USA) equipped with an electron capture detector (ECD) and a non-polar HP-5MS column (30 m × 0.25 mm i.d., 0.25 µm film thickness) and operated under the following conditions: column temperature was kept isothermal for 1 minute, then raised to 140 °C at a rate of 10 °C/min and maintained for a further 8 minutes isothermal, then increased again to 240 °C at a rate of 25 °C/min and finally remained isothermal for 5 minutes. Helium carrier gas flow rate was 1 mL/min and the temperature of the ECD was kept at 300 °C with a 60 mL/min N_2_ flow rate. The identification of acrylamide was performed by comparison of its’ retention time with that of the pure analytical standard (Sigma Aldrich, Steinheim, Germany). Matrix and reagent blanks, and a spiked material were simultaneously analysed to check the specificity and the recovery of the method used. Quantification of acrylamide was undertaken using a calibration curve of acrylamide in the concentration range from 0 to 6 ng/mL.

#### Determination of pesticide residues and polyaromatic hydrocarbons (PAHs)

For multi-residue pesticide analysis, the quick, easy, cheap, effective, rugged and safe (QuEChERS) method was used following the international standard [20]. The chromatographic analysis by GC-ECD of pesticide residues was performed using the same apparatus previously described after splitless injection. The separation of individual pesticides was carried out with the flowing temperature program: isothermal for 1 minute at 50 °C, increased to 100 °C at a rate of 25 °C /min, then to 300 °C at a rate of 5 °C/min, and kept isothermal for 5 minutes. The remaining analytical parameters (ECD temperature, carrier gas, flow rate) were kept as previously described. Pesticide residues were identified by comparison of their retention time with those of organophosphorus (Dichlorovos, ethoprophos, dissulfoton, methyl parathion, tokuthion), organochlorine (Aldrin, α-BHC, β-BHC, Dieldrin, Endrin, γ-BHC, heptachlor, heptachlor epoxide isomer B, 2,4’-DDD, 2,4’DDT, 4,4’ DDD, 4,4’DDE, 4,4’DDT), herbicide (Hexachlorocyclopentadiene, atrazine, simazine, alachlor, metolachlor, butachlor) standard mixtures and some individual analytical standard: α-endosulfane, endosulfane sulfate, bifenthrin, fenitrthion, cypermethrin (Sigma-Aldrich, Steinheim, German). A matrix blank, reagent blank and spiked material were simultaneously analysed to check the specificity and the recovery of the method. Once detected by GC-ECD, the presence of pesticide residues was confirmed by a Quattro Micro GC–MS system. The transfer line temperature was kept at 250 °C and the electron ionisation (EI) mode was 70 eV. The mass spectrometer was operating in selective ion monitoring mode (SIM). The confirmation of the presence of the suspect pesticide was based on the presence of at least 2 characteristic ions with ± 30% of the relative abundance previously determined for each reference material.

The solid-phase extraction (SPE) of PAHs was carried out using SPE cartridges containing a C_18_-silica hydrophobic sorbent and preconditioned with methanol and water (5 mL for each solvent) as previously described [21]. The PAHs chromatographic analysis was performed using a a Hewlett-Packard 6890 gas chromatograph (Agilent Technologies, Palo Alto, California, USA) equipped with a flame ionization detector (FID). Separation of individual PAHs was performed on a HP5-MS capillary column (30 m length × 0.25 mm i.d., · 0.25 µm film thickness) using the following gradient temperature: isothermal at 50 °C for 2 minutes, then increased at a rate of 8 °C/min to 300 °C and kept isothermal for a further 15 minutes. Temperatures of the injector and the FID detector were maintained at 270 and 320 °C, respectively. The flow rate of the carrier gas N_2_ was 1 mL/min and the split ratio was 10:1. The identification of individual PAHs was carried out by comparison of their retention times with those of commercial analytical standards: Phenantrene, acenaphtylene, acenaphtene, fluoranthene, pyrene, fluorine, naphthalene and benzonaphtalene (Sigma-Aldrich, Stenheim, Germany).

#### Statistical analysis

Results were expressed as mean ± standard error of at least three determinations. Comparison between means was performed by one-way analysis of variance (ANOVA) followed by Tukey’s Post-hoc test at the significance level of 5%. All analyses were performed using the Statistical Package for the Social Sciences (SPSS) software (version 18.0 for Windows, SPSS Inc., Chicago, IL, USA).

## Results and discussion

### Chemical analysis and mineral composition

Commercial chip samples made from potato and corn were analysed for their chemical composition and mineral content (Table 2). The average moisture, protein, fat and carbohydrate content were 1.16–3.59%, 1.31–6.11%, 30.29–42.98%, and 53.16–65.65%, respectively.

**Table 2:** Proximate composition (% on dry weight basis) of 13 commercial chips samples sold in the Tunisian market

Sample	Moisture	Proteins	Ash	Fat	Carbohydrates	Energy value (Kcal/100 g)
PTC1	3.29 ± 0.08^c^	4.03 ± 0.23^e^	0.62 ± 0.04^b^	31.43 ± 2.45^d^	65.65 ± 4.12^a^	561.58 ± 6.34^c^
PTC2	1.47 ± 0.12^ g^	2.27 ± 0.14^ g^	0.67 ± 0.06^b^	37.76 ± 1.12a^b^	60.55 ± 3.88^b^	591.11 ± 2.46^b^
PTC3	2.59 ± 0.26^d^	4.43 ± 0.36^d^	0.72 ± 0.06^ab^	37.1 ± 2.08^ab^	59.23 ± 4.36^b^	588.54 ± 4.69^b^
PTC4	1.16 ± 0.08^ h^	0.83 ± 0.04	0.66 ± 0.04^b^	42.98 ± 1.56^a^	57 ± 2.14^bc^	618.14 ± 6.71^a^
PTC5	3.19 ± 0.22^c^	6.11 ± 0.08^a^	0.64 ± 0.02^b^	30.29 ± 2.63^d^	64.51 ± 1.78^a^	555.1 ± 8.16^d^
PTC6	2.36 ± 0.18^d^	4.99 ± 0.28^c^	0.68 ± 0.02^b^	36.68 ± 0.84^c^	59.12 ± 2.28^b^	586.56 ± 6.55^b^
PTC7	2.58 ± 0.12^d^	1.31 ± 0.02^ h^	0.61 ± 0.02	37.51 ± 1.87^ab^	61.89 ± 3.67	590.34 ± 3.84^b^
PTC8	2.82 ± 0.16^d^	4.67 ± 0.22^d^	0.78 ± 0.04^a^	31.86 ± 0.18	64.68 ± 1.95^a^	564.15 ± 2.37^c^
CC1	1.98 ± 0.06^e^	3.87 ± 0.14^f^	0.54 ± 0.03	35.58 ± 0.08^c^	61.34 ± 3.47^b^	581.05 ± 5.66^b^
CC2	2.24 ± 0.14^de^	5.63 ± 0.08^b^	0.64 ± 0.03^b^	42.21 ± 3.82^a^	53.16 ± 2.32^c^	615.04 ± 8.41^a^
CC3	1.76 ± 0.06^f^	4.03 ± 0.21^e^	0.58 ± 0.02^c^	38.79 ± 2.43^a^	57.85 ± 1.62^bc^	596.64 ± 4.69^b^
CC4	3.8 ± 0.32^b^	4.19 ± 0.14^e^	0.68 ± 0.02^b^	38.1 ± 0.89^a^	58.32 ± 0.94b^c^	592.94 ± 4.37^b^
CC5	4.53 ± 0.26^a^	4.27 ± 0.36^d^	0.55 ± 0.04^c^	34.68 ± 1.77^c^	61.73 ± 1.44^b^	576.12 ± 3.85^b^

^*^Values within the same column followed by different superscript letters are significantly different at *p* < 0.05

The high carbohydrate content in all chip samples reflects the abundance in the original raw material (potato and corn) of these metabolites [22]. In contrast, the high fat content in all samples was primarily attributed to the oil uptake during the frying process, which in turn depends on time, temperature and quality of the frying oil [23]. The incorporation of oil and/or fat-rich ingredients (cheese, milk powder) during the formulation of chips could potentially increase the fat content in the end product [24]. In general, the obtained values were similar to those reported for Serbian potato chips (31.6–40.4%) [9], yet lower than those observed for the Peruvian potato chips [25]. The latter authors also found a negative correlation between fat content and moisture content, which is in line with the results obtained. Additional analyses indicated that all chip samples were found to have high energy values ranging from 555.1 to 618.14 kcal/100 g, representing the characteristic feature of chips as hyper-energetic products. The elevated energy value was primarily attributed to the abundance of high calories (fat and carbohydrates) versus low calories (proteins) in chip samples. This chemical pattern (high carbohydrate and fat level) in chips makes them particularly pleasurable to consumers. This is due to the production of a biochemical reaction that leads to the transmission of a pleasing message to the brain that induces a strong attraction and even an addiction to these hyper-energetic snacks. This message incites consumers to eat more even if they are not hungry, a phenomenon called as “hedonic hyperphagia” meaning “eating to excess for pleasure, rather than hunger,” [26]. This biochemical reaction allows a better understanding of the particular popularity of potato and corn chip snacks within all categories and ages of consumers [27]. Nevertheless, the high energy consumption could in turn represent a serious health concern due to their association with alteration of energy balance, leading to obesity and its associated diseases [28].

For ash content, the mean values were lower than 1% regardless of chip samples. This indicates that chip samples are a poor source of minerals. They are mainly composed of calcium (5.27–8.76 mg/g d.w), phosphorus (0.25–1.58 mg/g d.w) and magnesium (0.56–1.06 mg/g d.w) as the main macroelements, while zinc (0.22–0.41 mg/g d.w), iron (0.09–0.21 mg/g d.w), copper (0.01–0.032) and manganese (0.004–0.01 mg/g d.w) were the main microelements (Table 3). In general, this chemical pattern (low protein and mineral content, high carbohydrate and fat content) suggests that all chip samples are of low nutritive quality and confirm their appellation as “empty snacks” [29].

**Table 3:** Mineral content (mg/ g of dry matter) of 13 commercial chips samples sold in the Tunisian market

Sample	Macroelements			Microelements			
	Mg	P	Ca	Cu	Fe	Mn	Zn
PTC1	0.5582 ± 0.0723^e^	0.4551 ± 0.0595^f^	6.6255 ± 0.3459^b^	0.0162 ± 0.0007^f^	0.1038 ± 0.0166^d^	0.0040 ± 0.0006^f^	0.2772 ± 0.0561^c^
PTC2	0.5789 ± 0.0323^e^	0.8409 ± 0.0006^d^	5.2720 ± 0.8414^b^	0.0122 ± 0.0055^ g^	0.0971 ± 0.0269^d^	0.0047 ± 0.0006^f^	0.2199 ± 0.0604^e^
PTC3	0.6835 ± 0.0763^d^	0.8511 ± 0.0049^d^	5.8100 ± 0.9273^b^	0.0122 ± 0.0029^ g^	0.1122 ± 0.0063^d^	0.0053 ± 0.0004^f^	0.2820 ± 0.0459^b^
PTC4	1.0108 ± 0.6735^a^	0.4779 ± 0.0087^f^	8.7606 ± 1.9897^a^	0.0194 ± 0.0009^c^	0.1844 ± 0.0289^a^	0.0068 ± 0.0002^d^	0.3675 ± 0.0074^b^
PTC5	1.0611 ± 0.0122^a^	1.5839 ± 0.2047^a^	6.9619 ± 0.3042^b^	0.0181 ± 0.0023^d^	0.1562 ± 0.0631^b^	0.0535 ± 0.0256^a^	0.2890 ± 0.0357^a^
PTC6	0.7351 ± 0.1119^c^	0.8338 ± 0.0525^d^	7.1176 ± 3.4251^a^	0.0257 ± 0.0106^b^	0.2071 ± 0.1082^a^	0.0075 ± 0.0050^c^	0.4072 ± 0.2616^a^
PTC7	0.7582 ± 0.0079	1.0021 ± 0.0175^c^	9.6099 ± 2.2141^a^	0.0315 ± 0.0054^a^	0.2062 ± 0.0439^a^	0.0104 ± 0.0022^b^	0.3609 ± 0.2083^c^
PTC8	0.7453 ± 0.0026^c^	0.7824 ± 0.2231^e^	6.2906 ± 0.2018^b^	0.0147 ± 0.0003	0.0879 ± 0.0219	0.0029 ± 0.0010^ g^	0.2946 ± 0.1138^b^
CC1	0.6377 ± 0.0576^d^	0.8298 ± 0.1120^d^	8.0639 ± 0.8663^a^	0.0206 ± 0.0040^b^	0.1568 ± 0.0575^b^	0.0079 ± 0.0039^c^	0.2909 ± 0.0394^b^
CC2	0.7992 ± 0.1630^b^	0.6421 ± 0.1128^de^	6.6607 ± 2.5702^a^	0.0136 ± 0.0086	0.1384 ± 0.0586^c^	0.0061 ± 0.0029^d^	0.2681 ± 0.0905^c^
CC3	0.7795 ± 0.0636^b^	1.2146 ± 0.0699^b^	6.1677 ± 0.0961^b^	0.0319 ± 0.0242^a^	0.1243 ± 0.0020^c^	0.0083 ± 0.0005^c^	0.2937 ± 0.0114^b^
CC4	0.5609 ± 0.0188^e^	0.2478 ± 0.0185^ g^	6.0696 ± 0.9867^b^	0.0174 ± 0.0060^e^	0.1309 ± 0.0436^c^	0.0038 ± 0.0008^f^	0.3222 ± 0.0979^b^
CC5	0.8282 ± 0.2430^b^	0.7864 ± 0.0254^d^	8.2255 ± 1.0671^a^	0.0197 ± 0.0034^c^	0.1388 ± 0.0074^c^	0.0067 ± 0.0000^d^	0.2301 ± 0.0039^d^

^*^Values within the same column followed by different superscript letters are significantly different at *p* < 0.05

### Fatty acid composition and nutritional quality indices:

Gas chromatography analysis showed that saturated fatty acids (SFAs) contributed more than 39% of the total fatty acid profile except for CC3 and CC4 samples (Tables 4 and 5). Irrespective of chip samples, the SFA fraction was mainly dominated by palmitic acid (C16:0) which reached 45.38%, followed by myristic acid (C14:0) that reached 12.73% and stearic acid (C18:0) that rose to 4.84%. Oleic (C18:1) and linoleic (C18:2) acids were found as the main polyunsaturated fatty acids (PUFA), with amounts reaching 62.56% and 55.13% respectively for CC3 and CC4. For most of the samples, the fatty acid profile (C18:1 > C16:0 > C18:2) was consistent with that observed for potato and corn chips from Turkey [8]. Similar profiles were also described for potato and tortilla chips from Serbian origin [9]. However, some differences were observed in profiles of CC3 and CC4 samples, which exhibited significantly (p < 0.05) higher content of C18:2 and lower content of C16:0 fatty acids. It may be suggested that the different fatty acids profile of CC3 and CC4 can be attributed to their preparation method. This may be visibly more beneficial from nutritional point of view due to the use of a type or amount of oil in their formulation and/or frying.

**Table 4:** Fatty acid composition (% of total peak area) 13 commercial chips samples sold in the Tunisian market

	Fatty acid%							
Sample	C14:0	C16:0	C18:0	C18:1 cis- 9	C18:2 cis-9,12	C20:0	C18:3 cis-9,12,15	C22:0
PTC1	0.386 ± 0.003^c^	40.63 ± 3.21^b^	4.55 ± 0.22^a^	41.7 ± 3.46^b^	10.66 ± 1.72^c^	0.39 ± 0.006^a^	0.15 ± 0.004^b^	0.07 ± 0.0008^d^
PTC2	12.73 ± 0.17^a^	38.62 ± 2.81^b^	4.38 ± 0.30^a^	41.92 ± 1.86^b^	11.95 ± 1.27^c^	0.39 ± 0.006^a^	0.16 ± 0.006^b^	0.07 ± 0.0004^d^
PTC3	1.08 ± 0.01^e^	40.04 ± 2.38^b^	4.6 ± 0.21^a^	41.67 ± 2.97^b^	10.51 ± 1.75^c^	0.4 ± 0.003^a^	0.15 ± 0.005^b^	0.07 ± 0.0006^d^
PTC4	1.18 ± 0.02^e^	39.58 ± 0.94^b^	4.44 ± 0.33^a^	41.35 ± 3.06^b^	11.22 ± 1.43^c^	0.38 ± 0.003^a^	0.15 ± 0.006^b^	0.07 ± 0.0009^d^
PTC5	0.92 ± 0.01^f^	38.98 ± 3.73^b^	4.4 ± 0.20^a^	43.12 ± 1.64^b^	11.21 ± 1.66^c^	0.39 ± 0.008^a^	0.16 ± 0.005^b^	0.07 ± 0.0004^d^
PTC6	0.92 ± 0.009^f^	40.1 ± 3.77^b^	4.46 ± 0.28^a^	42.64 ± 3.00^b^	11.19 ± 1.04^c^	0.39 ± 0.005^a^	–	0.07 ± 0.0005^d^
PTC7	1.27 ± 0.02^e^	41.43 ± 3.37^b^	4.44 ± 0.34^a^	40.57 ± 3.04^b^	10.23 ± 1.49^c^	0.37 ± 0.007^a^	–	0.07 ± 0.0005^d^
PTC8	3.58 ± 0.04^c^	39.54 ± 2.04^b^	4.5 ± 0.35^a^	40.99 ± 1.94^b^	10.28 ± 0.45^c^	0.39 ± 0.009^a^	0.15 ± 0.005^b^	0.65 ± 0.008^c^
CC1	4.6 ± 0.06^b^	38.48 ± 3.78^b^	4.35 ± 0.16^a^	40.27 ± 3.31^b^	11.27 ± 0.84^c^	0.37 ± 0.005^a^	0.15 ± 0.002^b^	0.06 ± 0.0008^f^
CC2	1.35 ± 0.02^d^	39.49 ± 3.47^b^	4.56 ± 0.17^a^	41.07 ± 1.18^b^	10.63 ± 0.58^c^	0.38 ± 0.005^a^	–	0.06 ± 0.0007^f^
CC3	0.11 ± 0.003^f^	4.9 ± 3.49^c^	3.38 ± 0.18^b^	62.56 ± 0.5^a^	26.51 ± 1.81^b^	0.29 ± 0.008^b^	0.29 ± 0.009^a^	0.82 ± 0.008^a^
CC4	0.06 ± 0.006^ g^	6.74 ± 3.87^c^	3.6 ± 0.22^b^	33.73 ± 2.78^d^	55.13 ± 1.28^a^	–	–	0.73 ± 0.008^b^
CC5	1.42 ± 0.011^d^	45.38 ± 2.92^a^	4.84 ± 0.34^a^	37.96 ± 3.41^c^	8.13 ± 1.14^d^	0.39 ± 0.002^a^	–	0.07 ± 0.0007^d^

^*^Values within the same column followed by different superscript letters are significantly different at *p* < 0.05

**Table 5:** Lipid quality of 13 commercial chips samples sold in the Tunisian market

Sample	% SFA	%MUFA	% PUFA	%UFA	% cis	UFA/SFA	ω3	ω 6	ω3/ω6	AI	TI	Cox	Os
PTC1	46.03 ± 3.08^c^	41.7 ± 3.33^b^	10.81 ± 0.82^c^	52.51 ± 3.14^b^	52.5 ± 1.11^b^	1.14 ± 0.07^b^	0.15 ± 0.008^b^	10.66 ± 1.23^c^	0.01 ± 0.0014^a^	0.89 ± 0.08^d^	1.71 ± 0.05^c^	1.55 ± 0.06^c^	536.4 ± 6.29^f^
PTC2	56.19 ± 1.09^a^	41.92 ± 2.66^b^	12.11 ± 1.11^c^	54.03 ± 2.55^b^	54 ± 3.06^b^	0.96 ± 0.03^c^	0.16 ± 0.003^b^	11.95 ± 1.12^c^	0.01 ± 0.001^a^	1.74 ± 0.05^a^	2.03 ± 0.04^b^	1.68 ± 0.07^c^	595.7 ± 6.60^c^
PTC3	46.19 ± 2.41^c^	41.67 ± 1.71^b^	10.66 ± 0.82^c^	52.33 ± 2.67^b^	52.3 ± 3.23^b^	1.13 ± 0.03^b^	0.15 ± 0.004^b^	10.51 ± 1.04^c^	0.01 ± 0.001^a^	0.94 ± 0.07^d^	1.72 ± 0.08^c^	1.53 ± 0.04^c^	529.6 ± 7.24^f^
PTC4	45.65 ± 2.37^c^	41.35 ± 2.81^b^	11.37 ± 0.94^c^	52.72 ± 1.65^b^	52.7 ± 3.35^b^	1.15 ± 0.05^b^	0.15 ± 0.009^b^	11.22 ± 1.35^c^	0.01 ± 0.001^a^	0.92 ± 0.08^d^	1.69 ± 0.07^c^	1.60 ± 0.05^c^	561.3 ± 7.53^d^
PTC5	44.76 ± 1.93^c^	43.12 ± 1.66^b^	11.37 ± 1.09^c^	54.49 ± 3.50^b^	54.5 ± 1.46^b^	1.22 ± 0.06^b^	0.16 ± 0.008^b^	11.21 ± 0.67^c^	0.01 ± 0.0004^a^	0.86 ± 0.04^d^	1.60 ± 0.02^c^	1.62 ± 0.08^c^	563.6 ± 7.19^d^
PTC6	45.94 ± 2.09^c^	42.64 ± 1.58^b^	11.19 ± 1.35^c^	53.83 ± 3.27^b^	53.8 ± 2.82^b^	1.17 ± 0.05^b^	–	11.19 ± 1.25^c^	–	0.90 ± 0.03^d^	1.69 ± 0.02^c^	1.58 ± 0.05^c^	546.2 ± 6.17^e^
PTC7	47.58 ± 1.96^c^	40.57 ± 2.51^b^	10.23 ± 1.32^c^	50.8 ± 2.99^b^	50.8 ± 1.99^b^	1.07 ± 0.02^b^	–	10.23 ± 1.07^c^	–	1.00 ± 0.06^c^	1.86 ± 0.06^c^	1.46 ± 0.04^d^	500.9 ± 5.51^ h^
PTC8	48.66 ± 2.41^c^	40.99 ± 2.67^b^	10.43 ± 1.23^c^	51.42 ± 1.92^b^	51.4 ± 3.32^b^	1.06 ± 0.05^b^	0.15 ± 0.008^b^	10.28 ± 1.20^c^	0.01 ± 0.0019^a^	1.13 ± 0.08^b^	1.82 ± 0.07^c^	1.50 ± 0.06^d^	518.6 ± 7.73^ g^
CC1	47.86 ± 1.58^c^	40.27 ± 3.06^b^	11.42 ± 1.24^c^	51.69 ± 1.95^b^	51.7 ± 3.13^b^	1.08 ± 0.09^b^	0.15 ± 0.004^b^	11.27 ± 1.27^c^	0.01 ± 0.0013^a^	1.18 ± 0.07^b^	1.81 ± 0.04^c^	1.60 ± 0.04^c^	562.4 ± 8.26^d^
CC2	45.84 ± 1.77^c^	41.07 ± 3.64^b^	10.63 ± 0.90^c^	51.7 ± 3.65^b^	51.7 ± 3.47^b^	1.13 ± 0.07^b^	–	10.63 ± 1.30^c^	–	0.96 ± 0.05^ cd^	1.76 ± 0.06^c^	1.51 ± 0.09^d^	519.4 ± 7.67^ g^
CC3	9.5 ± 1.18^d^	62.56 ± 1.45^a^	26.8 ± 1.29^b^	89.36 ± 1.68^a^	89.4 ± 2.96^a^	9.41 ± 0.08^a^	0.29 ± 0.009^a^	26.51 ± 1.08^b^	0.01 ± 0.0008^a^	0.10 ± 0.02^d^	0.18 ± 0.08^d^	3.42 ± 0.06^b^	1285 ± 7.53^b^
CC4	11.13 ± 1.31^d^	33.73 ± 2.43^d^	55.13 ± 0.81^a^	88.86 ± 2.77^a^	88.9 ± 1.17^a^	7.98 ± 0.04^b^	–	55.13 ± 1.15a	–	0.12 ± 0.08^d^	0.23 ± 0.04^d^	6.02 ± 0.09^a^	2515 ± 6.37^a^
CC5	52.1 ± 0.85^b^	37.96 ± 3.51^c^	8.13 ± 1.23^d^	46.09 ± 3.49^c^	46.1 ± 3.08^a^	0.88 ± 0.07^c^	–	8.13 ± 0.96^d^	–	1.21 ± 0.08^b^	2.24 ± 0.09^a^	1.22 ± 0.06^e^	403.8 ± 7.61^i^

^*^Values within the same column followed by different superscript letters are significantly different at *p* < 0.05. *SFA* saturated fatty acids, *MUFA* monounsaturated fatty acids, *PUFA* polyunsatutrated fatty acids, *UFA* unsaturated fatty acids, *AI* atherogenic index, *TI* thrombogenic index, *Cox* calculated oxidizability, *OS* oxidative susceptibility

From a nutritional standpoint, the abundance of SFAs could be considered as a reliable indicator of the low nutritional lipid quality, as they are associated with the elevated risk of cardiovascular disease [9]. It was found that the high SFA versus low UFA content led to a low UFA/SFA ratio and high AI and TI. This indicates the poor lipid quality of all chip samples despite their low susceptibility to oxidation revealed by the low OS values (Table 5). Lower AI and TI values (0.81 and 0.54 respectively) were observed for the lipids extracted from of a wheat flour-based chips enriched with omega-3-rich fish oil. The high nutritional quality indices of the extracted lipids are due to the high amounts of essential PUFA in the enriched chips [30].

At this point, it can be suggested that corn and potato chips available in the Tunisian market have unfavorable fatty acid composition. It is advisable to reduce their consumption due to the possible increased incidence of obesity and cardiovascular related diseases.

### Acrylamide content

**Fig. 1:**
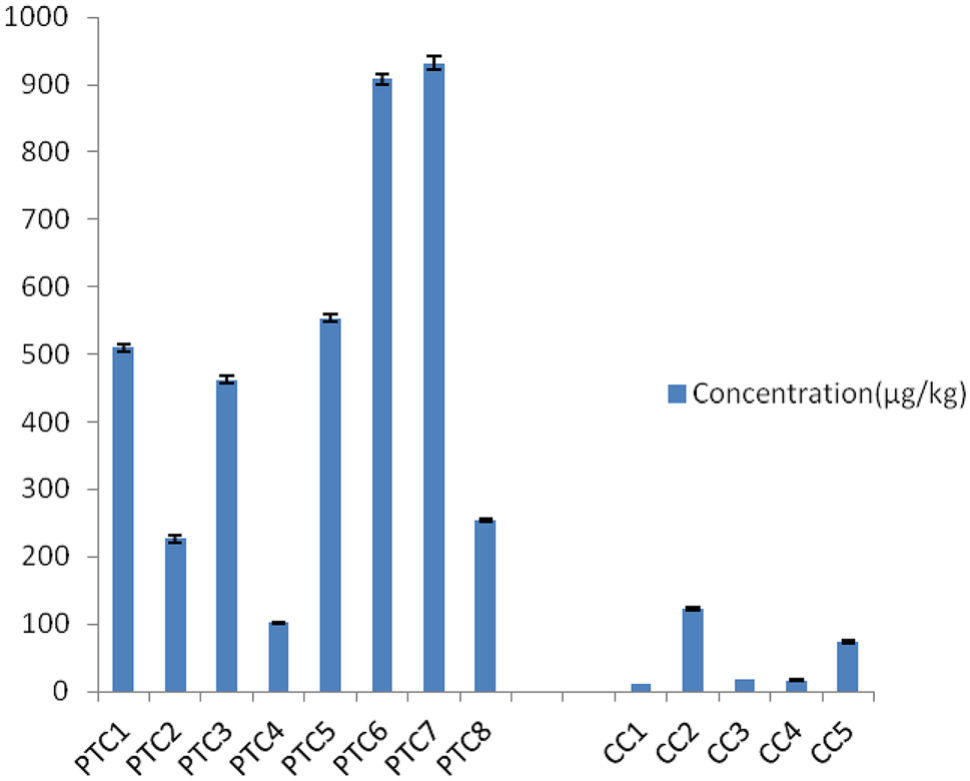
Acrylamide content of the chips samples

Gas chromatography analysis showed that all chip samples contained acrylamide, with a relatively high concentration exceeding for some samples (PTC6 and PTC7) the fixed threshold values in chips of 750 μg/kg (EU / 2017/2158) (Fig. 1). In general, potato chips contain more acrylamide than corn. In fact, the presence of acrylamide in chips was associated with the thermal action on asparagine and reducing sugars in cooked and fried foods. The more the food product is starchy, and the higher its sugar content, the more acrylamide that is formed [31–33]. These results are supported by literature data, which have shown the particularly high concentration of acrylamide in potato chips [34].

A significant variability of acrylamide level was also observed within the same type of chips (corn or potato).This can be explained by the fact that, in addition to the reducing sugar concentration, the final acrylamide level depends on many other factors. This include the processing conditions such as the frying temperature and time. Additionally this can be affected by the thickness of the chips, since the thicker they are the more they require heat input. Acrylamide content depends on the composition of the vegetable used, which could be affected by their origin, the annual variations of the agricultural conditions and their storage conditions [33].

Studies have shown that 0.052 and 0.064 mg/ kg bw/day are the average intake of acrylamide in potato chips for males and females, respectively [35]. Once ingested, acrylamide is rapidly absorbed and distributed in human organs [36]. Repeated consumption leads to its accumulation in organs [37].

Given its multi-organ carcinogenic and neurotoxic effects, the overconsumption of chips containing acrylamide could be associated with elevated risk of cancers, DNA damage, congenital anomalies and fetal malformations [36–38].

### Pesticide residues and PAHs content:

**Fig. 2:**
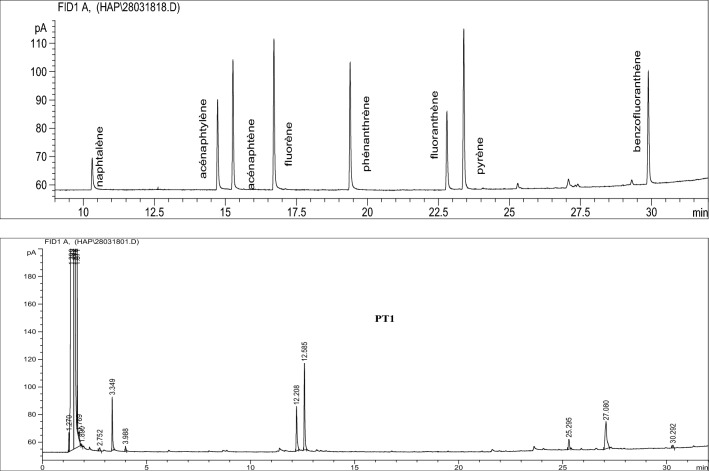
PTC1 chips sample chromatogram overlaid with PAHs mixture chromatogram

The majority of processed foods using physical and chemical methods, such as chips, may trigger the presence and/or the formation of mutagenic, genotoxic and carcinogenic substances such as pesticides and PAHs [39]. PAHs are organic contaminants that are known to be a major cause of cancer, their identified origins are fumigation, combustion of organic materials and heating [40]. The absence of PAH’s in the studied chip samples could be attributed to the absence of fumigation during the production process (Fig. 2). Unlike food processing conditions which are the major sources of PAH’s contamination, pesticides residues occurs from vegetable raw material, soil or water treatment. Excessive pesticide treatments during cultivation drive its persistence in food products in abundant amounts. Furthermore, it also leads to the contamination of the adjacent environment, including soil and water [41, 42]. Pesticide residues are known to be a major cause of birth defects, abnormalities of the central nervous system, endocrine disruption and cancer [43–45]

**Fig. 3:**
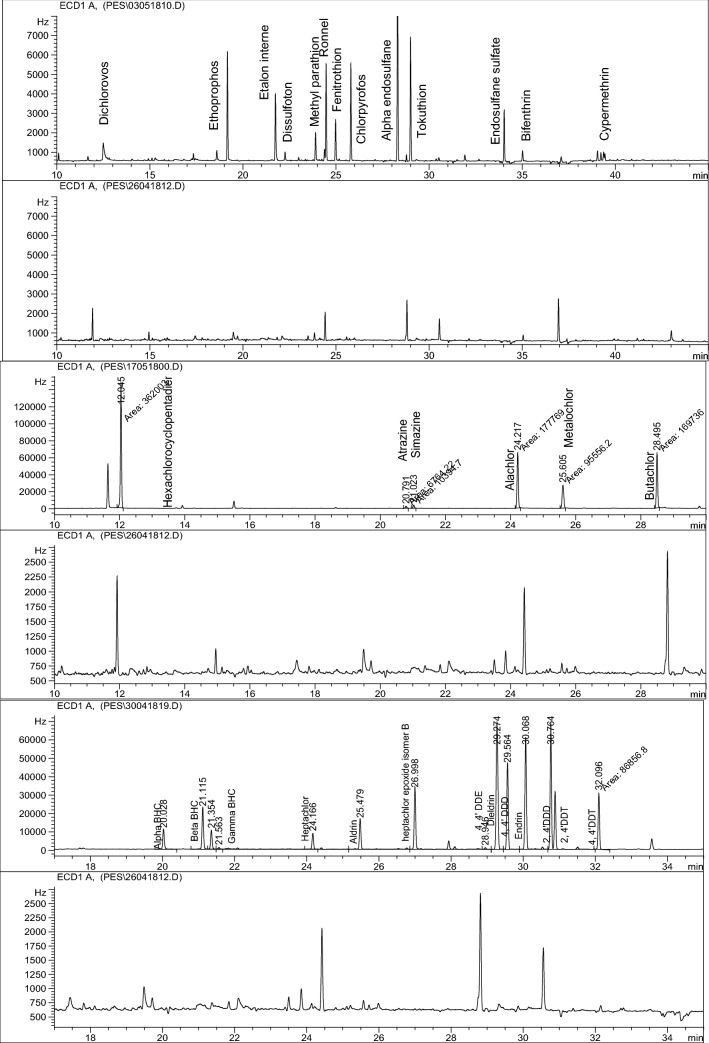
PTC1 chromatogram overlaid with pesticide mixtures chromatograms

Gas chromatography analysis did not show any detectable pesticide residues in the studied samples (Fig. 3). This indicates that the original raw material is devoid of pesticide residues.

It also could be suggested that PAHs and pesticides were eliminated during the manufacturing process. Generally, organic contaminant residues are much more abundant in the peels of potatoes then in the lashes [46, 47]. Consequently, the level of organic pollutants, such as pesticides and PAHs, is considerably reduced during the transformation, washing, and peeling process of vegetables. Removal of organic pollutant residues from potato varieties after peeling is possible; from 55.9% to 100%, and 57.5% to 100% for PAHs and pesticides respectively. The potential for transmission of contaminants from the peel to the lash strongly depend on the plant variety [48].

This hypothesis was confirmed by Soliman [49], who studied of the differences in the concentration of organochlorine pesticides in potato tubers, chips and fries, and found that the process of washing, peeling, bleaching and heating significantly reduced the concentration of pesticides.

## Conclusion

This study was carried out to determine the nutritional quality and safety of potato and corn based chips sold in the local Tunisian market. The results demonstrated the low nutritional value of the samples in terms of the high fat and carbohydrate content, and low protein and mineral content, which could be in the origin of many conditions, such as obesity and cardiovascular diseases. The study of the lipid quality indices of the samples highlighted the low nutritional lipid quality, evidenced by a high SFA versus a low UFA content, in addition to a high atherogenic (AI) and thrombogenic (TI) indices. Gas chromatography acrylamide analysis showed a high acrylamide content for most of the samples, with some exceeding the maximum residual limit set by the European Commission, therefore indicating a potential risk to the consumers’ health. Nevertheless, analysis of contaminants, such as pesticide residues and PAHs within the chip samples, did not show any safety concerns..

To conclude, this study highlighted the poor nutritional value of the chips sold in the Tunisian market, additionally suggesting the need to design novel functional snacks fortified with bioactive compounds and nutraceuticals beneficial for health and well-being.

## Data Availability

The dataset generated for this study are available on request to the corresponding author.
